# Elevated serum 25-hydroxy (OH) vitamin D levels are associated with risk of TB progression in Gambian adults

**DOI:** 10.1016/j.tube.2016.02.007

**Published:** 2016-05

**Authors:** Olumuyiwa Owolabi, Schadrac Agbla, Patrick Owiafe, Simon Donkor, Toyin Togun, Abdou K. Sillah, Martin O.C. Ota, Jayne S. Sutherland

**Affiliations:** a Vaccines & Immunity Theme, Medical Research Council Unit, Atlantic Road, Fajara, Gambia; b Department of Medical Laboratory Science, University of Health and Allied Sciences, PMB 31 Ho, Volta Region, Ghana; c World Health Organisation Regional Office, Brazzaville, Congo

**Keywords:** Tuberculosis, Incident cases, 25(OH) D, Vitamin D binding protein, Latent TB infection

## Abstract

**Background:**

Vitamin D is essential in the host defence against tuberculosis (TB) as an immune modulator. The aim of this study was to determine the level of 25-hydroxyvitamin D (25 (OH) D) from adult TB index cases before and after treatment and their exposed household contacts (HHC) in The Gambia.

**Methods:**

Serum from adult index TB cases and their TB-exposed household contacts (HHC) was analysed for 25(OH) D and Vitamin D binding protein (VDBP) concentrations. Tuberculin skin test (TST) status was used as a measure of *Mycobacterium tuberculosis* (Mtb) infectivity in the HHC. In addition, HHC who later progressed to active TB (incident cases) were assessed alongside non-progressors to determine the influence of 25 (OH) D levels on TB risk.

**Results:**

Eighty-three TB cases, 46 TST+ and 52 TST− HHC were analysed. Generally levels of 25(OH) D were considered insufficient in all subjects. However, median levels of 25(OH) D and VDBP were significantly higher in TB cases compared to both TST+ and TST− HHC at recruitment and were significantly reduced after TB therapy (p < 0.0001 for all). In addition, levels of serum 25(OH) D at recruitment were significantly higher in TB progressors compared to non-progressors (median (IQR): 25.0(20.8–29.2) in progressors and 20.3 (16.3–24.6) ng/ml in non-progressors; p = 0.007).

**Conclusion:**

In The Gambia, an equatorial country, 25(OH) D levels are higher in serum of TB progressors and those with active disease compared to latently infected and uninfected subjects. These results contrast to findings in non-equatorial countries.

## 1 Introduction

The global burden of Tuberculosis (TB) is huge, with an estimated one-third of the world population latently infected [1], [2], 9 million new cases and 1.5 million deaths per year [3]. In a life-time, 10% of infected individuals progress to active TB disease in immune competent individuals increasing to 10% per year in HIV co-infected individuals [4]. One of the major contributing factors to disease progression in HIV uninfected individuals is malnutrition [5] with micronutrient deficiencies such as vitamins A, B6, E, thiamine, folate, and zinc reported in patients with active TB disease [6]. 25-hydroxyvitamin D (25(OH) D) has been implicated in the host defence against TB as an immune modulator [1], [7], [8]. Indeed, treatment of TB decades ago included exposure to the sun for enhanced 25(OH) D synthesis, but the mechanism was not clear [9]. 25(OH) D has been shown to down-regulate the pro-inflammatory response and therefore may help to protect the host against increased lung pathology induced by exacerbated inflammation [10]. It also has anti-microbial effects by inducing activation of macrophages [11]. A seminal paper in Science demonstrated the link between 25(OH) D and IFN-γ induced macrophage antimicrobial responses [12].

The majority of studies analysing the role of 25(OH) D in TB have shown an association between low 25(OH) D and susceptibility to active TB disease [13], [14], [15], [16]. However, there are still discrepancies in terms of genetic predisposition [17], [18] and serum levels in TB and non-TB subjects [19], [20]. This is likely due to geographic location, exposure to sunlight, genetic differences, cultural practices, religion and the presence of other disease conditions [21]. A recent study in Greenland, for example, showed large variation of Serum 25(OH) D concentrations in TB patients, with supplementation likely to increase the risk of TB amongst those with normal or high concentrations [22]. Indeed, clinical trials in Guinea-Bissau and Georgia and a meta-analysis of all trials that used 25(OH) D supplementation as adjunctive therapy have shown no clinical benefits [23], [24], [25]. In HIV-infected patients in Uganda, no difference in 25(OH) D levels was seen in subjects with and without TB [23], while a Malawi study among TB patients with over 50% HIV co-infection rates showed that deficiency in the levels of 25(OH) D was common and had no influence on response to treatment [27].

The genetic predisposition to TB associated with Vitamin D receptor (VDR) gene polymorphisms was considered a prime candidate for TB susceptibility but results from a meta-analysis have proved inconclusive [17] although a link between VDR polymorphisms and response to therapy in terms of time to fast sputum conversion rate with anti TB treatment in FF; Taql Tt and non-FF; TT genotypes respectively for pulmonary TB has been suggested [24]. These discrepancies are further highlighted by a recent study showing no benefit of 25(OH) D supplementation on response to therapy in an Indian population [25] although it may limit lung pathology and thus reduce disability-associated life-years (DALYs) in vulnerable populations [26].

In West Africa, there appears to be a role for VDR haplotypes rather than genotypes in susceptibility to TB [18], although another study in Guinea Bissau showed VDR polymorphisms when analysed together with ethnicity were associated with increased risk of TB disease [27]. Interestingly, there also appears to be an influence of variation within the gene encoding Vitamin D binding protein (VDBP) and Serum 25(OH) D levels in Gambian children [28], which has implications for interpretation of 25(OH) D status across different groups. However, no study has been performed in The Gambia to determine the serum levels of 25(OH) D in TB patients, TST+ and TST− contacts.

Due to our powerful TB case-contact platform in The Gambia [29], we are able to analyse individuals across the spectrum of TB infection and disease. Thus, the aim of this study was to compare the level of Serum 25(OH) D and VDBP in adult TB cases before and after treatment and their exposed household contacts (both TST+ and TST−). Importantly, we also analysed exposed subjects who progressed to active TB disease between 3 and 24 months from recruitment. This is the first study in Gambia, an equatorial country, to determine 25(OH) D levels and association with TB. In complete contrast to the majority of studies in non-equatorial countries, we found higher 25(OH) D levels in TB cases compared to TST+ and TST− HHC. Importantly, levels decreased with treatment and were higher in subjects who progressed to active TB, indicating that in our setting higher 25(OH) D is associated with TB risk.

## 2 Materials and methods

### 2.1 Study participants and design

Adult TB cases were recruited from the Medical Research Council (MRC) TB clinic in Fajara, The Gambia. The diagnosis of active TB was established on the basis of smear positivity for acid-fast bacilli of *Mycobacterium tuberculosis* (Mtb). All mycobacterial cultures were identified and confirmed using standard procedures. Household contacts (HHC) of confirmed index cases were visited to assess their TB infection status and were followed for 2 years from recruitment. Tuberculin skin test (TST) was used as a measure of mycobacterial infection and was performed using 2 tuberculin units of purified peptide derivative RT23 (SSI, Denmark) injected intra dermally into the volar aspect of the forearm and read at 48–72 h. TST was performed prior to any blood sampling of study participants. A reading of ≥10 mm was considered positive, and indicative of mycobacterial infection. The diagnosis of latent TB infection was established on the basis of absence of TB symptoms, absence of radiologic abnormalities of active TB (all contacts had chest X-ray performed) and positivity for TST.

25(OH) D was measured at recruitment and 6 months after recruitment for adult TB cases but measured only at recruitment for TST+ and TST− HHC. The VDBP was measured for a random subsample of TB cases, TST+ and TST− HHC (n = 24 cases at 0 and 6 months; 27 TST+ HHC and 28 TST− HHC). These had similar age and sex distributions to the larger cohort (not shown). A matched case–control study was also conducted to analyse TB progressors (defined as asymptomatic at recruitment but progressing to active disease between 3 and 24 months [29] and matched (by age and sex)) to non-progressors in a 1:3 ratio. Serum samples from all subjects were collected and stored at −20 °C until analysis. All participants in this study gave written informed consent.

### 2.2 Measurement of 25(OH) D levels

Serum 25(OH) D concentrations were determined using a Vitamin D total ELISA (DIAsource Immunoassays, Belgium) according to the manufacturer's instructions. A 4-parameter logistic function curve was used to determine the 25(OH) D concentrations of the samples from the calibration curve. 25(OH) D levels were defined as: Deficiency: 0–10 ng/ml; Insufficiency: 10–30 ng/ml; Sufficiency: 30–150 ng/ml and toxicity: >150 ng/ml.

### 2.3 Measurement of vitamin D binding protein levels

In order to determine the free, bioactive levels of 25(OH) D, we analysed Serum Vitamin D binding protein (VDBP) using an ELISA-based method (Immundiagnostik, Germany) according to manufacturer's instructions. A 4-parameter standard curve was constructed and levels of VDBP determined in each sample. Results were multiplied by 40,000 and converted to mg/dl.

### 2.4 Statistical analysis

The 25(OH) D level at recruitment was compared between TB groups using Wald adjusted test accounting for clustering within households. Unadjusted and adjusted mixed effects models were performed with age, gender and season at recruitment as potential confounders. We investigated the relationship between 25(OH) D and BMI using a multilevel structural equation modelling accounting for clustering within households. Wilcoxon signed rank test and Wald adjusted test were used to compare the 25(OH) D and VDBP levels between TB groups at 0 and 6 months. Pearson chi-squared test with second-order correction of Rao and Scott accounting for clustering within households was used to assess association between TB groups and categorical variables such as gender and 25(OH) D category (i.e. insufficient versus sufficient) at recruitment. Wald test with adjustment for confounders using linear regression was used to analyse progressors and non-progressors.

## 3 Results

### 3.1 Participant demographics

We analysed a total of 181 HIV sero-negative participants consisting of 83 confirmed TB cases, 46 TST+ HHC and 52 TST− HHC (Table 1). A total of 12 incident cases (progressing to active TB between 3 and 24 months from recruitment) were detected and matched by age and sex with 32 HHC who did not progress to active TB. There was no difference in age between TB cases, TST+ HHC and TST− HHC but a significant difference in the proportion of males in the TB index case group (71.1%), TST+ (52.2%) and TST− (36.5%) HHC groups (p = 0.002). As expected, BMI was significantly lower in TB cases compared to both TST+ and TST− HHC (median (IQR) for TB cases was 17.6 (16.3–19.4) compared to 20.6 (19.1–22.7) and 20.3 (18.7–23.6) kg/m^2^ for TST+ and TST− HHC respectively) (Table 1). Due to differences in age and sex and possible household clustering, all results have been adjusted for these variables.

### 3.2 Serum 25(OH) vitamin D levels

**Figure 1:**
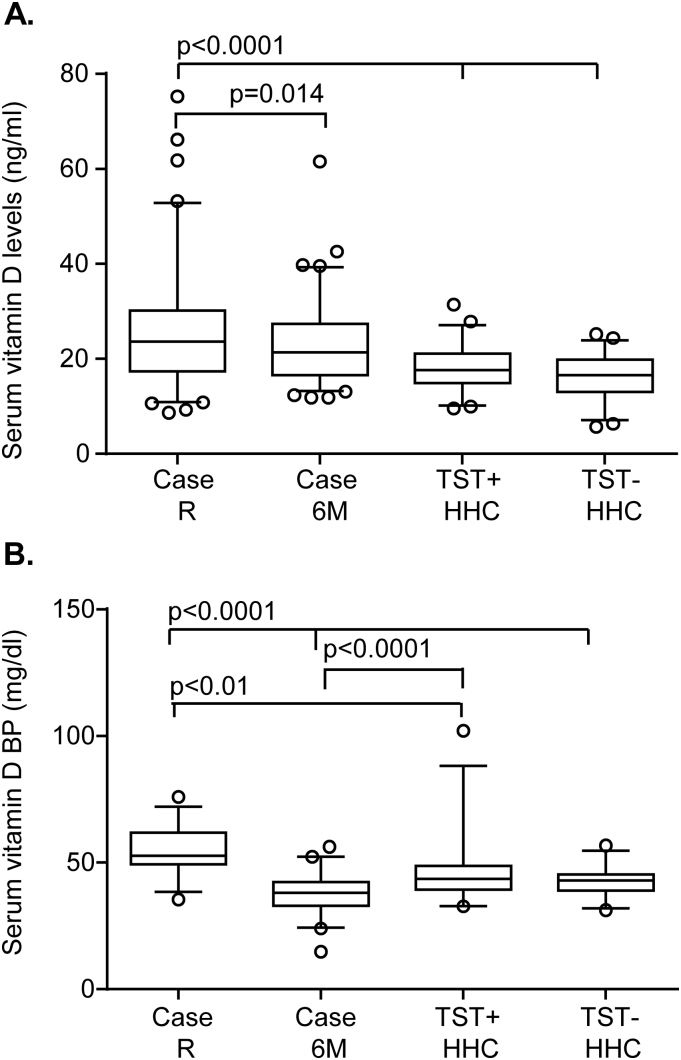
25(OH) D levels in TB cases and household contacts (HHC). A: Serum 25(OH) D levels were measured using ELISA. Box plots (5–95%) are shown for Serum 25(OH) D levels of TB cases at recruitment (R) and 6 months of treatment and TST+ and TST− HHC at recruitment. Line indicates median and dots indicate outliers. B: Serum Vitamin D Binding Protein levels were measured using ELISA. Box plots (5–95%) are shown for TB cases pre and post-treatment compared to TST+ and TST− HHC. Data were analysed using Random effects modelling and Wilcoxon Ranked Sums test (for comparison of TB cases at recruitment and 6 months). A p-value ≤ 0.05 was considered significant.

**Figure 2:**
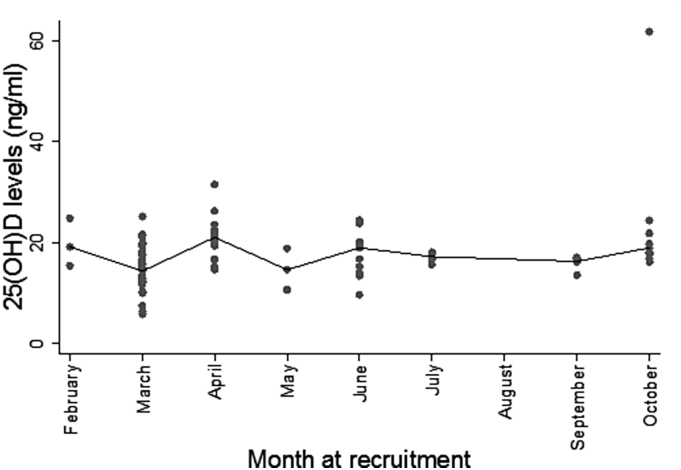
Median 25(OH) D levels of TB cases and Household contacts (HHC) at recruitment. Median 25(OH) D levels at each month of recruitment for all participants are shown to reflect changes in levels of 25(OH) D levels.

26.5% of TB cases had sufficient levels (30–150 ng/ml) of 25(OH) D compared to 4.3% of TST+ HHC and 0% TST− HHC (p = 0.0001), while 2.4% of TB cases, 4.3% TST+ HHC and 5.8% of TST− HHC had deficient levels according to the Endocrinology Society [30]. The median (interquartile range (IQR)) 25(OH) D levels were significantly higher in TB cases pre-treatment compared to both the TST+ and TST− HHC (23.6 (17.1–30.3), 17.4 (14.9–21.1) and 16.3 (13–19.6) ng/ml respectively; p < 0.0001 for both; Figure 1a). Following successful TB treatment, 25(OH) D levels significantly decreased from pre-treatment to post-treatment levels (median (IQR): 23.6 (17.1–30.3), 21.3 (16.6–27.3); p = 0.02; Figure 1a) respectively, but were still significantly higher than the HHC (p = 0.007 for TST+ and p < 0.0001 for TST−). We used a multivariable linear regression model to adjust for age, gender, household clustering and season at recruitment. None of these variables had any confounding effects on the relationship between TB groups and 25(OH) D at recruitment (Table 2). Levels remained relatively constant throughout the year with a slight but not significant dip in March (Figure 2). However, this was adjusted for and had no confounding effects on the relationship between TB groups.

We did not include BMI as potential confounder because BMI falls into the causal pathway for TB. However, we investigated whether BMI acted as a mediator between TB groups and 25(OH) D levels using a multilevel structural equation modelling. There was no evidence of mediation effect of BMI on the relationship between TB group and 25(OH) D level, adjusted for age, gender and season at recruitment (data not shown).

### 3.3 Analysis of vitamin D binding protein levels

In order to determine if there were differences in the functional component of 25(OH) D, we analysed Vitamin D binding protein (VDBP), measured on a random sub-sample of TB cases (n = 24), TST+ HHC (n = 27) and TST− HHC (n = 28). These subjects had similar age and sex distribution compared to the larger cohort (not shown). VDBP showed the same pattern as for total 25(OH) D with respect to TB status: TB cases had significantly higher levels of VDBP compared to both TST+ and TST− HHC (median (IQR) = 52.6 (49.2–61.7), 43.4 (39.5–48.7) and 42.8 (39.1–45.3) mg/dl respectively; p = 0.01 compared to TST+ and p < 0.0001 compared to TST− HHC; Figure 1b). Additionally, levels were significantly reduced in TB cases following treatment (median (IQR): 38.0 (33.0–42.3) mg/dl; p < 0.0001 compared to pre-treatment levels) and were comparable to TST− HHC (p = 0.25) but significantly lower than TST+ HHC (p = 0.02; Figure 1b).

### 3.4 Increased 25(OH) D levels in incident TB cases

The median (IQR) 25(OH) D level at recruitment was significantly higher at recruitment for the incident cases (progressors) (25.0 (20.8–29.2) ng/ml) compared to the non-progressors (20.3 (16.3–24.6) ng/ml; p = 0.0006; Table 1). The 25(OH) D was higher in progressors than non-progressors (95% CI: 1.20, 7.53; p = 0.007) after adjusting for season at recruitment (Table 3).

## 4 Discussion

25(OH) D has been implicated in the immune response to Mtb infection by influencing the inflammatory response and subsequent anti-microbial immunity [12]. This has led to the hypothesis that 25(OH) D is important in the control of Mtb infection and therefore individuals with insufficient levels are more susceptible to disease. However, in our study, we found the converse: patients with active TB had insufficient but significantly higher levels of 25(OH) D and VDBP compared to asymptomatic controls and this level was reduced by 6 months of anti-TB therapy. Furthermore, household contacts that progressed to active disease within 3–24 months had higher levels of 25(OH) D at recruitment than matched controls that did not progress. These findings suggest that higher levels of 25(OH) D than in the general population might be a risk factor for TB disease progression in Gambian adults.

Currently, there is little data available on 25(OH) D and susceptibility to infections in West Africa. In addition, while polymorphisms in the VDR have been seen in Gambians (for example the genotype tt (suggesting resistance to clinical TB) were underrepresented in adult TB cases samples compared with controls), these could not be definitively linked to TB susceptibility [31]. Interestingly, the majority of studies demonstrating insufficient 25(OH) D in TB patients have been performed in countries with distinct summer and winter seasons and thus differences in levels of natural sunlight. The only other study we found that showed similar results to ours was in Uganda, suggesting that there could be geographical differences in the immunological effects of 25(OH) D. Importantly, our results show no confounding with season, gender or age.

We did not assess the downstream production of the active form of 1, 25-dihydroxy D (1, 25(OH) 2 Ds). However, analysis of 25(OH) D is preferable to assess nutritional Vitamin D status and to diagnose 25(OH) D deficiency and insufficiency while 1,25(OH)2 D is more applicable for correlation with VDR polymorphisms [32]. The increase in 25(OH) D levels in asymptomatic subjects who later progressed to active TB might suggests that elevated 25(OH) D is a risk factor for TB disease progression in our setting and that supplementation may be detrimental.

The limitations of our study were a lack of mechanistic insight, insufficient powering and absence of longitudinal measurements in the contacts. The mechanisms of action of 25(OH) D are complex and diverse [33] and should be analysed in conjunction with VDR polymorphisms. Future work should include mechanistic approaches such as *in vitro* manipulation with Vitamin D compounds to assess anti-microbial immunity in blood from Gambian TB patients. In addition, our findings by ELISA should be confirmed by mass spectrometry, which is superior for measuring 25(OH) D levels.

It is plausible that the differences we see in 25(OH) D levels in active TB disease in Gambians is a contributing factor to the underlying immune differences between West Africans and other geographically-diverse subject: for example IFN-γ production following Mtb antigen stimulation in studies involving five different sub-Saharan African countries [34]. We did not analyse 25(OH) D in relation to disease severity, particularly cavitary disease by chest x-ray, but it would be of interest to see if Gambian subjects have reduced levels of TB lung-sequelae compared to patients from other countries with reduced 25(OH) D. A larger cohort powered to assess lung function before and after therapy would be beneficial.

In conclusion, insufficient 25(OH) levels are not a risk factor for TB disease in HIV uninfected Gambian adults. Conversely, elevated 25(OH) D appears to be a risk factor for progression to active disease. Our findings suggest that Vitamin D supplementation is unlikely to be beneficial.

## Figures and Tables

**Table 1:** Demographic characteristics, nutritional status and vitamin D level for the study participants.

	TB Index case (n = 83)	TST+ HHC (n = 46)	TST− HHC (n = 52)	p-Value	TB incident case (n = 12)	Non-progressors (n = 32)	p-Value
Age, median (IQR))	28 (23–39)	24 (22–33)	23 (20–35)	0.08∗	30.5 (21–56)	26 (20–49)	0.39‡
Male, n (%)	59 (71.1)	24 (52.2)	19 (36.5)	0.002†	8 (66.7)	23 (71.9)	0.74§
BMI at recruitment, median (IQR)	17.6 (16.3–19.4)	20.6 (19.1–22.7)	20.3 (18.7–23.6)	<0.0001∗	20.7 (17.7–21.4)	21.4 (19.7–24.7)	0.07‡
Vitamin D level, median (IQR)	23.6 (17.1–30.3)	17.4 (14.9–21.1)	16.3 (13.0–19.6)	<0.0001∗	25.0 (20.8–29.2)	20.3 (16.2–24.6)	0.006‡
*Vitamin D category at recruitment*							
Sufficiency (30–150 ng/ml), n (%)	22 (26.5)	2 (4.3)	0 (0.0)		3 (25.0)	1 (3.1)	
Insufficiency (10–30 ng/ml), n (%)	59 (71.1)	42 (91.3)	49 (94.2)		9 (75.0)	31 (96.9)	
Deficiency (0–10 ng/ml), n (%)	2 (2.4)	2 (4.3)	3 (5.8)		0 (0.0)	0 (0.0)	

Age and sex were used to match TB incident cases to non-progressors.

∗ Wald adjusted test accounting for clustering within households.

† Pearson chi-squared test with second-order correction of Rao and Scott accounting for clustering within households.

‡ Wald test from mixed effects linear regression accounting for matched-pairs clustering.

§ Wald test from mixed effects logistic regression accounting for matched-pairs clustering.

**Table 2:** Estimated effects of TB group, age, gender and season at recruitment on Vitamin D level at recruitment.

Variables	Unadjusted effect (95% CI)∗	p	Adjusted effect (95% CI)∗	p
TB group		<0.0001†		<0.0001†
Active TB	0		0	
TST+ HHC	−7.20 (−10.9, −3.52)	<0.0001	−7.02 (−10.7, −3.33)	0.001
TST− HHC	−9.72 (−13.0, 6.46)	<0.0001	−9.14 (−12.6, −5.7)	<0.0001
Age	−0.004 (−0.12, 0.11)	0.95	−0.07 (−0.17, 0.04)	0.20
Gender				
Female	0		0	
Male	4.87 (1.66, 8.07)	0.003	2.38 (−0.55, 5.32)	0.11
Season at recruitment				
Dry	0		0	
Wet	−1.92 (−6.40, 2.55)	0.40	−0.53 (−4.42, 3.37)	0.79

∗ 95% Confidence intervals.

† Joint Wald test assessing overall evidence association between TB group and vitamin D level. Other p-values are also derived from Wald test assessing the significance of the estimated effects. For continuous and binary variables, Wald tests assess both significance of estimates and overall evidence of association with vitamin D level.

**Table 3:** Unadjusted and adjusted association between vitamin D level and TB progressors, adjusted for season at recruitment.

Variables	Unadjusted effect (95% CI)∗	p†	Adjusted effect (95% CI)	p†
Incident cases				
Non-progressor	0		0	
Progressor	4.21 (1.31, 7.13)	0.005	3.65 (0.59, 6.71)	0.02
Season at recruitment				
Dry			0	
Wet			1.72 (−1.47, 4.92)	0.29

∗ 95% Confidence intervals.

† Wald test.
